# Exploring Institutional Change in the Context of a Statewide Developmental Education Reform in Florida

**DOI:** 10.1080/10668926.2019.1610672

**Published:** 2019-05-02

**Authors:** Christine G. Mokher, Hayley Spencer, Toby J. Park, Shouping Hu

**Affiliations:** Educational Leadership & Policy Studies, Florida State University, Tallahassee, Florida, USA

## Abstract

We explore institutional change and organizational learning in higher education in the context of a large-scale reform of developmental education in Florida. We use statewide survey data to examine administrators’ perceptions of the use and effectiveness of accountability metrics, methods to identify solutions to improve student outcomes, and challenges responding to data. We find that institutions most commonly use accountability data to track their own performance, but find it less effective for making comparisons across colleges. Institutions use a variety of methods to identify solutions for improvement; with the most common method being presentations at professional association conferences. The greatest challenges in reviewing and responding to data are finding resources to implement solutions and understanding underlying problems affecting student outcomes. We conclude with implications for policymakers and state agency staff designing large-scale reforms in order to encourage organizational learning and promote meaningful change.

Institutional change in higher education can be very difficult to implement, particularly when large-scale changes must occur quickly due to a new policy, mandate, or piece of legislation. Policy development in higher education tends to occur as incremental change over time instead of rapid and large-scale reforms (Mintrom & Norman, 2013). Deviating from the typical method of implementing changes can make rapid, large-scale reforms more difficult and can increase the amount of resistance from those affected by these changes. This resistance stems from institutions and individuals that feel pressured to preserve the status quo and many of the difficulties associated with making large-scale changes. The larger the scale of a change and the more people and institutions it involves, the harder it is for all parties affected to coordinate changes at the institutional level, particularly for those with different motivation, capacity, and work settings (Leithwood, Jantzi, & Mascall, 2002).

Accountability systems are one mechanism intended to improve institutional effectiveness. Accountability systems play a role in the institutional change in a variety of ways, but an increasingly common method among public colleges is the use of performance funding. The intention of using performance funding is to incentivize institutions to improve student outcomes by tying a portion of appropriations to different performance indicators. While the use of performance funding as an incentive is widely used in higher education, research has not demonstrated a clear indication of its effectiveness. For instance, Dougherty et al. (2016) found little evidence that institutions can respond effectively. The few published studies that have found some positive effects tend to be part of broader mixed results. For example, Tandberg and Hillman (2013) found that few states demonstrate positive gains after adopting performance funding policies, and in the few states that did have a positive effect, the effects did not become evident for at least seven years. Additionally, Hillman, Hicklin Fryar, and Crespín-Trujillo (2018) found that while there were modest positive effects of performance-based funding on certificate completion in Ohio and Tennessee, there were no effects on associate or bachelor’s degree completion rates. This limited increase in student momentum as a result of performance funding may occur because there is little evidence that institutions can build the capacity to implement changes to react to new accountability metrics (Dougherty et al., 2016; Hillman, Tandberg, & Fryar, 2015). This lack of capacity may occur for many different reasons ranging from inadequate financial resources, to a lack of understanding of the underlying institutional problems that impact performance, to an inability to identify how to address those problems effectively. For accountability systems to function as a means to improve student outcomes, the capacity of institutions to make changes should be understood and addressed.

Increasing the role of accountability systems can influence the speed and perception of institutional change. Little is known about how organizational learning and institutional change occur in higher education (Kezar, 2005). As Huber (1991) noted, there are multiple ways for knowledge to be acquired by organizations, but that for organizational learning to truly occur the knowledge must be distributed, interpreted, and committed to memory. However, even if organizational learning occurs, it does not always lead to observable changes or improvements in institutional effectiveness (Huber, 1991). Institutional change can occur apart from organizational learning, however, understanding the influences of organizational learning on institutional change is an important part of the change process in higher education.

Since little is known about how organizational learning and institutional change occur in higher education, examining these processes during a statewide reform can provide valuable context and insight. In 2013 the Florida legislature passed Senate Bill 1720 (SB 1720) which required the 28 public institutions in the Florida College System (FCS) to implement a statewide reform of developmental education programs. Developmental education programs include a series of courses in math, reading, and writing designed to improve students’ academic preparation. Prior to fall 2014, students enrolling at one of the 28 institutions were required to enroll in developmental education courses, unless they received college-ready scores on the placement test which would allow them to enroll directly in higher level courses that may count towards college credit. The reform included making developmental education courses optional for many students through the exemption of placement tests for high school graduates entering a public Florida high school in 2003/04 or later, and active duty military personnel. Students who were not eligible for the exemption were required to enroll in developmental education or provide college-ready test scores to enroll in non-developmental education courses.

For students still enrolling in developmental education, the courses were required to be offered as contextualized, modularized, compressed, or co-requisite modalities. Contextualized courses offer instruction related to different academic programs with similar foundational skills. Modularized instruction provides smaller portions of content, typically through computerized modules, for students to focus on building only the skills where they have deficits. Compressed instruction offers the courses in a shorter timeframe to reduce the time spent in developmental education. Co-requisite courses occur at the same time as a credit-bearing course to allow developmental education to supplement the credit-bearing course. Through this legislation, large-scale changes occurred quickly with full implementation of the reform beginning in fall 2014.

With every institution in the Florida College System being affected by SB 1720, the scale of coordination would have been immense if a single entity was responsible for managing implementation across all institutions. Instead, institutions were responsible for making decisions (such as which modes of developmental education courses to offer) and implementing those changes, which allowed for differences by the institution in how organizational learning occurred throughout this change process. By the spring of 2014, each institution was required to prepare an implementation plan and submit it to the Division of Florida Colleges that indicated the decisions made about the redesign of instructional strategies, the implementation of a more involved advising process, and the provision of additional support services available to all students. These plans revealed that institutions had a variety of unique plans for reforming institutional practices, which tended to be grounded in researched-based promising programs and practices (Hu, Tandberg, Nix, Collins, & Hankerson, 2014). These plans also informed the use of data related to reform initiatives, as the colleges were required to describe their provisions for the collection of student success data to facilitate analysis and identification of successful strategies.

Additionally, the level of accountability increased under the reform as the legislation required new accountability reports to be completed annually to examine changes in student outcomes following the policy implementation (SB 1720, 2013). These Developmental Education Accountability Reports allow the state to monitor progress on indicators such as grade distributions and enrollment rates for each of the modalities of developmental education courses. Data were collected from institutions using the Department of Education’s Florida‘s PK-20 Education Information Portal (EdStats) online business intelligence tool to create a consistent accountability report across all institutions. This report was prepared annually in accordance with section (s.) 1008.30(6)(b), Florida Statutes (FS) by the Florida College System, and submitted to the governor, the state legislature, and the State Board of Education (Florida College System, 2017).

Beginning in 2016, the Florida legislature established a new FCS Performance-Based Incentive Program, which further monitored institutional progress on student outcomes. This program was codified under s.1001.66, Florida Statutes, and tied a portion of state funding to institutions’ progress on four longer-term outcomes of retention rates, completion rates, job placement/continuing education rates, and entry-level wages (Florida College System, 2018). Performance on these metrics was used to place each college into one of four categories that reflects its total points relative to the system mean and standard deviation. The Florida Department of Education uses this information from the performance funding system to prepare reports that allow the findings to be easily interpreted and compared across institutions.

The purpose of this study is to observe institutional change processes at the Florida College System (FCS) institutions following the developmental education reform under the implementation of SB 1720. This study builds on previous annual surveys of administrator perceptions conducted by the Center for Postsecondary Success since the reform began in 2014. While previous surveys explored the types of changes made as a result of the implementation, this survey seeks to gain more detail about the processes of institutional change. We utilize two conceptual frameworks to guide our research. The first is Huber’s (1991) five process for understanding the knowledge acquisition process, which provides insight into how institutions identified solutions to implement under the reform. The second is Dougherty et al.’s (2016) conceptualization of four mechanisms through which accountability metrics may influence institutional change. This allows us to explore how accountability metrics that were implemented by the state, both directly addressing developmental education and more broadly accounting for longer-term student success, may have influenced institutional decision-making during the reform. These frameworks led to the following research questions:

1. What are college leaders’ perceptions of the use and effectiveness of accountability metrics under SB 1720?
2. What methods do college leaders use to identify solutions for improving outcomes?
3. What challenges do college leaders face in reviewing and responding to data?

These questions were examined through a system-wide survey of senior administrators’ perceptions following the implementation and effects of the developmental education reform on institutional change at the 28 colleges. The survey focused on how institutions have used data over time to make changes and how accountability metrics from the legislation are perceived. This study contributes to the areas of organizational learning and institutional change by examining these processes in the context of the implementation of the large-scale developmental education reform in Florida.

## Literature review

As our research questions focus on the role of accountability metrics and organizational learning through institutional change, the literature provides connections to these topic areas. Organizational learning can provide insight into how institutions acquire knowledge to make meaningful change, including utilizing data available from accountability metrics. Thus, we begin by utilizing a conceptual framework by Huber (1991) to explore how institutions may have acquired knowledge that informed their decision-making in the reform’s implementation. Next, we examine the role of accountability metrics in institutional change. We use a second conceptual framework by Dougherty et al. (2016) to examine how institutional decision-making under the reform may have been further influenced by the statewide Developmental Education Accountability Reports and the FCS Performance-Based Incentive Program. This literature base has important implications for understanding the organizational learning and institutional change processes that occurred in the developmental education reform in Florida, and these implications will be explored in this study.

### Organizational learning processes

When large-scale reforms (such as SB 1720) are implemented, institutions use organizational knowledge to create institutional change. Both structural and cultural shifts may need to occur to allow for new ways of knowledge acquisition. In this study, we use a conceptual framework by Huber (1991) to explore five ways in which knowledge can be acquired during the organizational learning process. The first method is congenital learning, or knowledge passed down from the founders of the organization, which influences past and future processes in the organization. Experiential learning is the second method, which occurs with direct experimentation of different ideas. Third is vicarious learning, or the process through which members learn second-hand from others’ experiences. The fourth method is grafting, which is when knowledge from new organizational members is transferred to the existing organization. The final method of knowledge acquisition is searching and saving, which involves the organization actively identifying solutions from within the organization or from the external environment. We posit that institutions may have used any of these methods to acquire knowledge to inform their decisions about how to implement the reform, either alone or in combination. For example, an institution could learn second-hand about another institution’s experience implementing co-requisite developmental education by talking with other staff at that institution (vicarious learning) and then pilot that modality at their own institution to see how it works before deciding whether to bring it to scale (experiential learning).

In addition to the five methods of knowledge acquisition, there are different learning processes that organizations undergo when identifying and solving issues. Argyris (1976) describes single-loop learning as correcting mistakes without attempting to solve underlying issues, and double-loop learning as going back to the organizational structure and culture to understand and address the underlying issues found in the single-loop process. While double-loop learning can provide more effective solutions, organizations often have difficulties finding the time and resources to complete this process. This may help to explain why accountability practices do not always lead to increased organizational learning and institutional change. While accountability can increase the collection and use of data, double loop learning will not occur if organizations do not attempt to make meaningful changes using data (Hora, Bouwma-Gearhart, & Park, 2017). We further explore these processes in the context of Florida’s developmental education reform by asking survey respondents about how challenging they find it to understand underlying problems affecting student outcomes and to identify effective solutions to address these problems when reviewing and responding to data on student outcomes.

### Role of accountability metrics in institutional change

Accountability has become increasingly common in higher education as a means of ensuring that institutions are meeting the expectations of both students and the state. However, it may be difficult to develop appropriate accountability metrics if policymakers do not fully understand what factors influence student outcomes or face difficulties in operationalizing the metrics to hold institutions accountable for these factors. For example, Horn and Lee (2017) found that indicators typically used in accountability reports were not consistently able to predict institutions that would meet certain performance measurements. Yet despite issues in using accountability to drive performance, the use of these types of incentive programs is increasing.

The second framework that we use to guide our research is Dougherty et al.’s (2016) conceptualization of the four mechanisms through which organizational behavior may be influenced by accountability metrics. The first mechanism is the use of financial incentives to motivate institutions to improve student outcomes in order to maximize the revenues available through performance funding. The second mechanism is societal expectations, which can put pressure on institutions to make behavioral changes to improve performance. The third mechanism is status striving, which is the process through which institutions learn more about their student outcomes and attempt to improve their performance to be on par with peer institutions, typically out of pride. The last mechanism identified is the process of organizational learning, which can occur when institutions identify weak areas and find new approaches to improve their performance. However, this may occur infrequently in practice, as there is little evidence that institutions can build capacity and respond effectively to incentives from performance funding. Institutions may not be able to develop feasible and effective solutions or understand the underlying problems associated with poor performance.

We examine the extent to which these four mechanisms may have further influenced institutional decision-making under Florida’s developmental education reform, as there were two different types of accountability mechanisms being utilized by the state during this time. The first type was the FCS Annual Developmental Education Accountability Reports, which were closely aligned with the goals of the reform. There was no funding associated with this report, so the financial incentive mechanism would not be relevant. However, these reports may have influenced institutional outcomes by other mechanisms such as societal expectations and status striving if institutional leaders perceived that their college was ineffective at improving developmental education outcomes, particularly relative to other colleges. The second type of accountability during this time was the FCS Performance-Based Incentive Program, which was not specific to the reform but looked more broadly at institutional performance on longer-term student outcomes like credential completion. This could potentially complicate institutions’ response to the developmental education reform if they encounter pressure to focus on other metrics, particularly given the presence of financial incentives in this program.

## Methods

### Survey design

We utilized a statewide survey of lead administrators at FCS institutions to understand the process of institutional change in the context of Florida’s developmental education reform. The questions used to answer our research questions were included in a larger survey that consisted of 21 questions addressing topics such as the process of data use, types of institutional changes and their perceived influences on student outcomes, and perceptions of institutional performance. The questions examined in this study examined challenges in reviewing and responding to data, methods of identifying solutions, and perceptions of the Developmental Education Accountability Report and Performance-Based Incentive Program. There were 16 multiple choice questions, which often included a series of sub-questions such as rating the effectiveness of accountability programs on multiple dimensions. Most of these multiple-choice questions were measured on a five-point scale, such as not at all (1) to a great extent (5), strongly disagree (1) to strongly agree (5), and not at all effective (1) to most effective (5). In addition, five open-ended questions were included in the survey that asked respondents to provide examples of changes in emphasis on programs the college made in response to the Performance-Based Incentive Program, ways that incoming students’ preparation affects accountability metrics, additional data they would like to have on student outcomes, and actions taken by institutions seen as leaders in improving outcomes.

More specifically, the survey questions analyzed in this study focused on the extent to which administrators reported that their college used different methods to find solutions that improve student outcomes (e.g. attending presentations at meetings or conferences by professional associations, visiting other colleges to learn about their approaches, etc.). These questions were included in the survey as we wanted to understand how institutions obtain knowledge to solve problems or improve student outcomes, based on Huber’s (1991) framework of knowledge acquisition. Understanding how institutions acquire knowledge provides insight into the organizational learning process.

Additionally, administrators were asked how they use data from the FCS Annual Accountability Report on student success in relation to developmental education, and how the effectiveness of these reports were perceived. These questions were included to understand the relationship and the perceptions of accountability metrics and how data was used by institutions, based on Dougherty et al.’s (2016) conceptualization of accountability processes. Lastly, questions about the effectiveness of the FCS Performance-Based Incentive program, established in 2016 by the Florida legislature, were asked. These questions included administrators’ perceptions of the effectiveness of the program, the extent to which the college’s emphasis on different programs or services has changed as a result of the program, and the perceptions of the extent to which incoming students’ academic preparation affects the accountability metrics. These questions were included as performance-based funding may influence institutional change by incentivizing improvement in priority areas. We also examine the extent to which these priority areas were complementary to the goals of the developmental education reform.

### Survey administration

Utilizing the 28 FCS institutions’ websites to identify senior administrators in academic and student affairs, we found email addresses for multiple individuals from all colleges. Each identified administrator received an email with a link to the survey, which was administered online using Qualtrics software. Administrators were instructed to work with their colleagues to generate one response per institution. The survey was administered in the spring semester of 2018, with initial distribution occurring in the first week of April 2018. The survey remained open for one month to allow administrators to collaborate where necessary to answer all survey questions.

### Sample

The response rate for the survey was 68%, with administrators at 19 of the 28 FCS colleges completing the survey. One institution had two respondents complete the survey, so in this case, the response from the most senior-level administrator was used. Respondents included provosts or vice presidents (63%), deans (16%), and other administrators involved in institutional effectiveness or strategic planning (21%).

### Data analysis

A comprehensive descriptive analysis of the data from the survey was conducted utilizing Stata. Based on the survey questions, results were organized into four categories: use of data on student outcomes, types of institutional changes made in response to data, administrators’ perceptions of accountability metrics, and assessment of institutional performance in improving student outcomes. For closed-ended questions, we tabulated responses across the scale and created figures to represent the results. Open-ended questions were analyzed to find emerging themes in the responses, as well as to identify information providing further context into a respondent’s closed-ended responses.

## Results

### Perceptions of the use and effectiveness of accountability metrics

**Figure 1.**
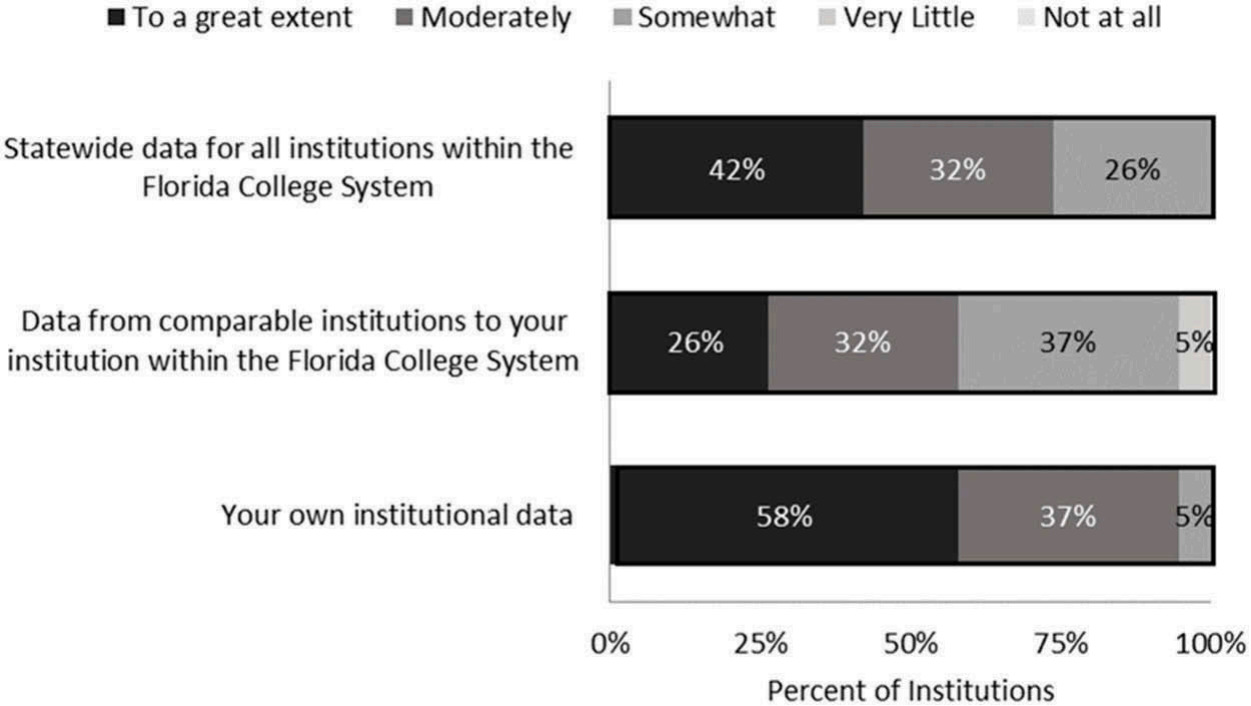
Extent to which colleges monitored various indicators on the Developmental Education Accountability Reports. Note: Scale ranges from 1 (not at all) to 5 (to a great extent). N = 19 institutions.

**Figure 2.**
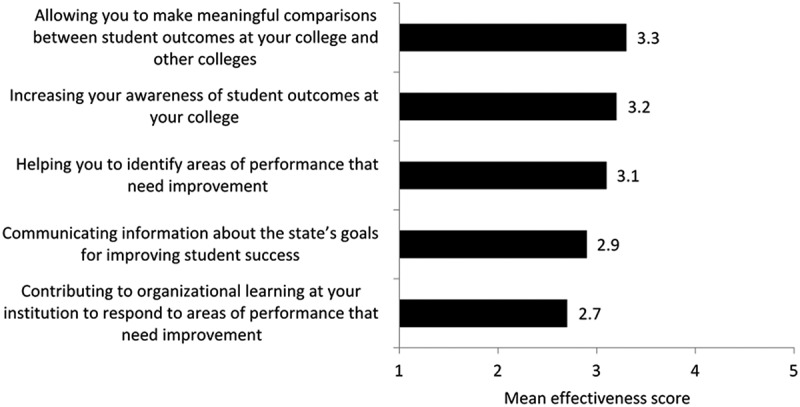
Mean effectiveness score for uses of the Developmental Education Accountability reports. Note: Scale ranges from 1 (not at all effective) to 5 (most effective). N = 19 institutions.

The results of the survey indicate that institutions primarily use accountability data from the Developmental Education Accountability Reports to track their performance and statewide data, with three-quarters or more of respondents indicating that they used the data “moderately” or “to a great extent” for these purposes (Figure 1). In comparison, just over half (58%) of institutions reporting using the Developmental Education Accountability Report to the same extent to track the performance of comparable institutions in the Florida College System. This indicates data were used less frequently to make comparisons among peers. Additionally, administrators perceive these accountability data to be moderately effective at allowing them to make meaningful comparisons across other colleges, with a mean effectiveness rating of 3.3 on a 5-point scale (Figure 2). The lower effectiveness ratings could be because institutional leaders do not perceive the data to be comparable across colleges. As one respondent noted:

Each state college in Florida prepares a Developmental Education Accountability report that is due at the end of October. The first report required schools to focus on “two at-risk populations.” Institutions did not all use the same lens in selecting populations. Some used FTIC other used the total student population, and not every institution breaks down by race or gender.

Interestingly, these data are perceived to be less effective in contributing to organizational learning to respond to areas that need improvement, with a mean effectiveness rating of 2.7. The average effectiveness rating across all indicators was less than 4.0, indicating that the Developmental Education Accountability Report was not perceived to be “very” or “most” effective at any of its purposes. This suggests there is room to improve the effectiveness of the different uses of this report.

**Figure 3.**
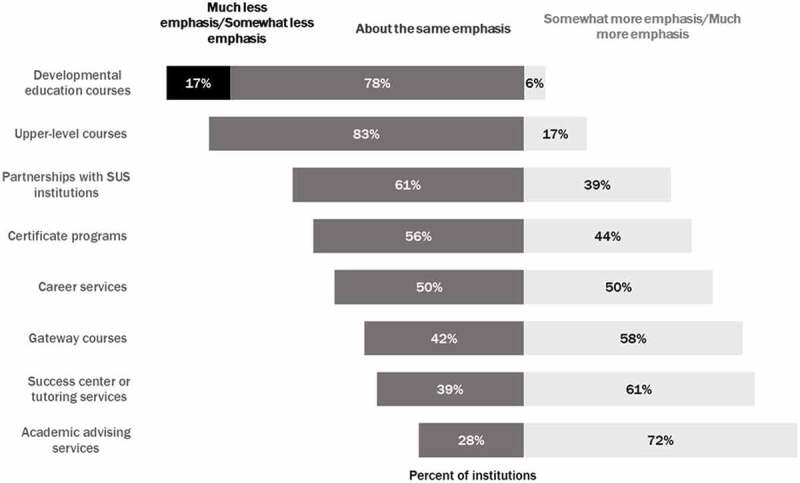
Extent to which institutional leaders think their college’s emphasis has changed for various activities in response to the Performance-Based Incentive Program. Note: Scale ranges from 1 (much less emphasis) to 5 (much more emphasis). N = 19 institutions.

The Performance-Based Incentive Program also appeared to influence the amount of emphasis that institutions placed on different priorities. Figure 3 displays these changes and shows that developmental education was the only area where institutions reported less emphasis as a result of this program. Seventeen percent of institutions reported placing “much less” or “somewhat less” emphasis on developmental education, while 78% reported that the emphasis remained the same. The areas with the greatest gains in emphasis were success centers or tutoring services (61%) and academic advising services (72%). With the majority of students becoming exempt from placement testing and developmental education under SB1720, these two areas could be more active in helping students that would have been placed in developmental education courses prior to the reform. The same trends were echoed in the open-ended responses. One respondent noted:

Performance based incentives had a large effect on tutoring services, as we know tutoring is an effective strategy. We expanded the tutoring centers, added more tutors, implemented embedded tutors in gateway courses and other courses with low pass rates, and expanded outreach efforts to inform students about tutoring and academic support services. We also expanded efforts to track students receiving tutoring services and evaluating the penetration levels in targeted courses for the number and percent of students attending tutoring sessions.

This perception was shared by another respondent who said:

Performance based incentives had a large effect on advising services, as advisors spend more time gathering and analyzing student data prior to advising sessions, spend more time with students in advising appointments, and spend more time tracking student progress and working with struggling students.

**Figure 4.**
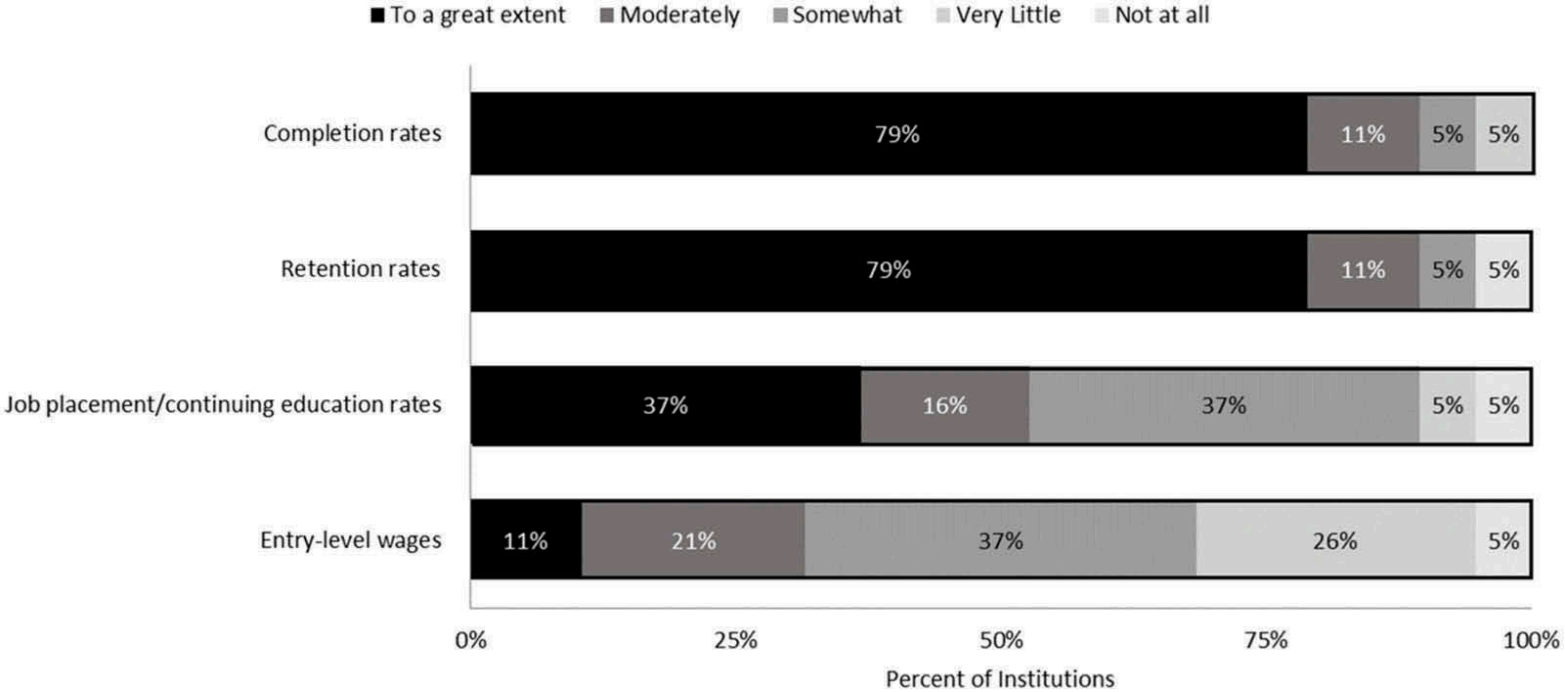
Extent to which college leaders perceive the academic preparation of their incoming students affects various metrics in the Performance Based Incentive Program. Note: Scale ranges from 1 (not at all) to 5 (to a great extent). N = 19 institutions.

The developmental education reform also has implications for how institutional leaders perceive their performance on accountability metrics, since students with weak academic preparation are no longer required to enroll in developmental education. Ninety percent of institutional leaders perceived retention rates and completion rates, two accountability metrics used in the Performance-Based Incentive Program, as being affected “moderately” or “to a great extent” by incoming students’ academic preparation (Figure 4). Responses to the open-ended portion of this question focused mainly on incoming students’ lack of preparation affecting retention and completion rates. As one respondent noted:

It is more difficult to retain students who are not academically prepared from semester to semester and year to year. Students who are not academically prepared often lack the social and academic capital to stay in school and complete their program of study.

Another respondent indicated:

As a result of performance-based funding, we have placed great emphasis on FTIC students and ensuring their retention from fall to fall. Given that many students tend to enroll in Gateway courses during their first semester or first year, these courses have been given more attention.

Several institutions also reported that the Performance-Based Incentive Program led them to increase their emphasis on certificates and applied associate degree programs since these credentials are also recognized in the new accountability metrics. One administrator explained that his or her institution was “continually reviewing and modifying AS programs with embedded college-credit certificates to ensure that we are preparing students well for the workforce.” Another administrator reiterated this emphasis on career and technical education programs, noting:

Our recent focus has been with individual businesses and industry associations to “strike a deal” that we will provide the workforce training they need if they will a) hire our students as interns and b) allow them, even encourage them, to finish the program so that the student has the basis for a successful career.

Thus, institutions may be subject to goal conflict if they are facing pressure to decrease their emphasis on developmental education even though longer-term outcomes are being influenced by underprepared students. Interestingly, entry-level wages and job placement were not perceived as being affected to the same extent as retention rates and completion rates. This could possibly be due to the timing of these metrics occurring after students are retained and have completed their programs of study.

### Methods used by college leaders to identify solutions for improving outcomes

**Figure 5.**
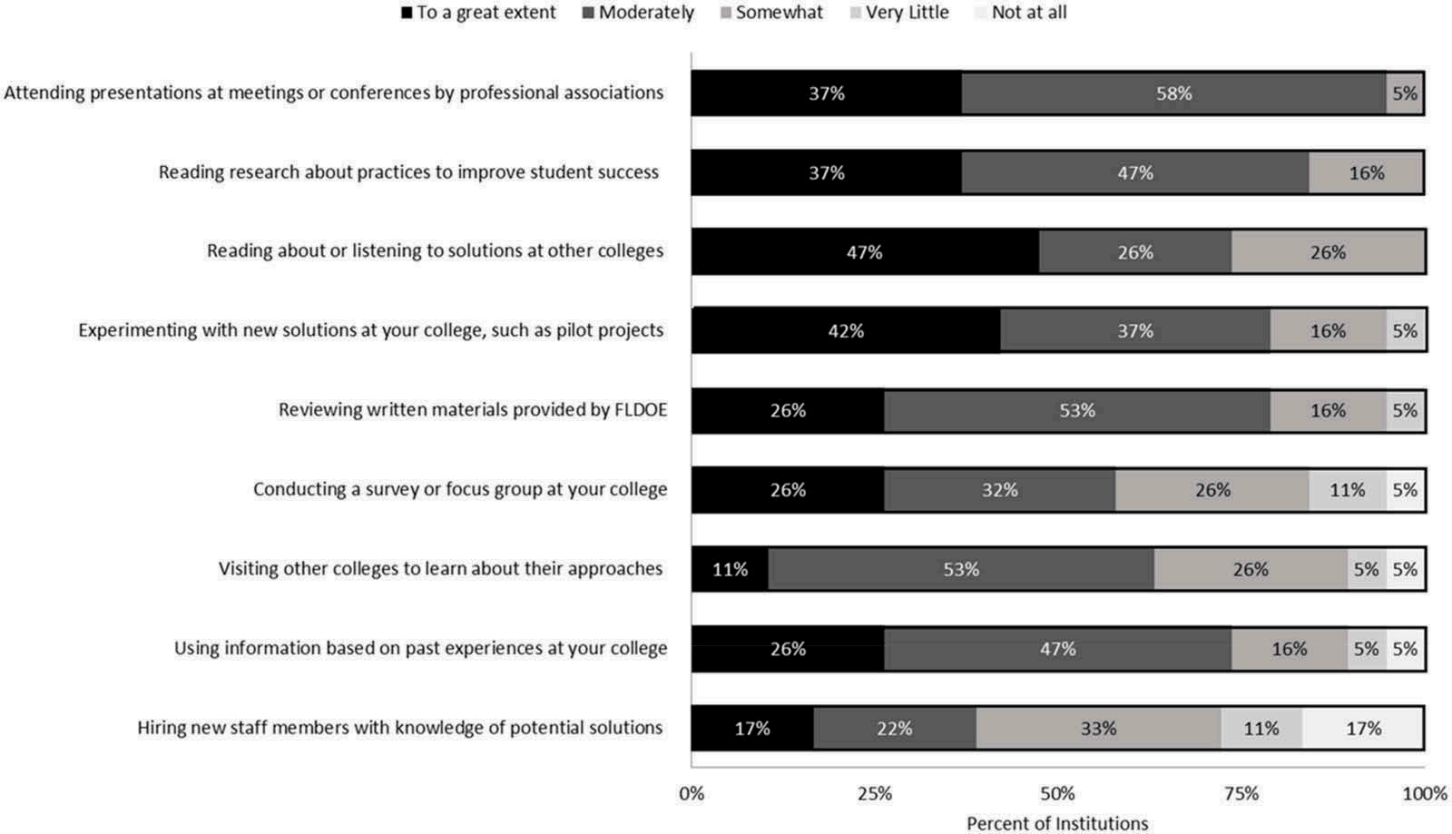
Extent to which colleges used various methods for identifying solutions for improving student outcomes. Note: Scale ranges from 1(not at all) to 5 (to a great extent). N = 18 to 19 institutions, depending on the sub-question.

Figure 5 contributes to answering our second research question on how institutions identify solutions to improve student outcomes. As shown from the results, the most common methods included some form of professional development or networking to determine what practices were working best for other institutions. Ninety-five percent of respondents reported “moderately” or “to a great extent” identifying solutions from attending presentations at meetings or conferences by professional associations, while 84% reported reading research about practices to improve student success. Slightly less common were institutions learning from their own experiences, with 79% experimenting with new solutions like pilot projects at their college, and 73% using information based on past experience at their college. The least commonly used methods were those that were time and resource-intensive such as hiring new staff members with knowledge of potential solutions (39%responded that they used this method “moderately” or “to a great extent”), which aligns with the responses showing that finding resources is the most challenging issue when reviewing and responding to data.

### Challenges faced by college leaders in reviewing and responding to data

**Figure 6.**
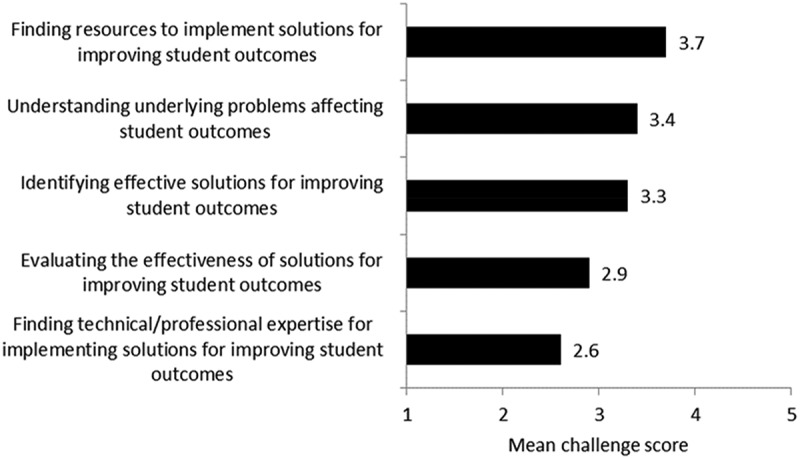
Mean score for how challenging various issues is when reviewing and responding to data on student outcomes. Note: Scale ranges from 1 (not challenging at all) to 5 (extremely challenging). N = 19 institutions.

Even if institutions have access to data on their performance and have discovered methods for identifying solutions, they may still face challenges in determining how to respond. Leaders were asked to rate on a scale of 1 (not at all challenging) to 5 (extremely challenging) how challenging various issues are when responding to data on student outcomes. Finding resources to implement solutions was perceived as the greatest challenge in reviewing and responding to data, with a mean score of 3.7 (Figure 6). Other major challenges for institutions included understanding underlying problems affecting student outcomes (mean = 3.4) and identifying effective solutions for improving student outcomes (mean = 3.3), both of which would also require additional time and resources to solve. As one administrator noted:

While we have expended considerable resources in advising, academic success centers, career centers, and early alert systems, we are seeing ongoing and increasing concerns about exempt student readiness in English and math gateway courses. We have not seen evidence at the institutional or state level to verify that SB1720 facilitates the path to completion or supports higher levels of student success. Instead, we see a growing number and percentage of incoming exempt FTICs who struggle in their first term, despite greater levels of advising, tutoring, and student support.

Several institutions also reported in the open-ended responses that they would like to have additional data to inform decision-making that is not currently collected as part of the accountability metrics. For example, several respondents indicated that the data currently collected presents snapshots at a single point in time, which makes it difficult to monitor progress longitudinally both across cohorts and within the same cohort over time. As one administrator noted, “we need long-term data on the effect of placing students in developmental courses that could possibly be successful in college-credit bearing courses. Additionally, [we need] progression data on STEM and Non-STEM pathways.*”* Another respondent indicated that faculty needed better access to their own course data including success rates. There was also a call for the use of more data provided directly by students. As one administrator noted, “qualitative data, such as student stories will be helpful as numbers alone do not tell the entire story.*”* Another administrator expressed a desire for a way to standardize and centrally collect data on instructor ratings from student course evaluations in developmental and gateway courses. The availability of these additional types of data may provide a more complete picture of the underlying problems contributing to student success rates, which may allow leaders to respond more effectively.

## Discussion

The responses from the survey provide a better understanding of institutional change occurring after a statewide reform of developmental education. Overall, our findings indicate that institutions have taken vastly different approaches to the institutional change process under SB 1720. Yet all institutions continue to adapt over time; responding by using data in decision-making processes, learning from their own experiences, and exploring innovations undertaken by others.

We used Dougherty et al.’s (2016) conceptualization of accountability mechanisms to understand how statewide accountability may influence the institutional change process in the context of the developmental education reform. Institutions tend to frequently use their own data and statewide data from the Developmental Education Accountability Reports to inform the changes made in response to the reform, which suggests that the mechanism of organizational learning may be prevalent. Institutions are less likely to use the data to make comparisons among peer institutions, indicating that the status-striving mechanism is less common. There could be many reasons for these differences, but one may play into another very important policy occurring alongside the reform, performance-based funding. The metrics aligned with the Performance-Based Funding Initiative may provide more emphasis on institutions comparing themselves with statewide metrics as opposed to only the comparable institutions in the Florida College System. These metrics may also encourage some institutions to place less emphasis on developmental education than they otherwise would have, and focus more on services perceived to influence longer-term outcomes like degree completion such as enhanced student support services. This could contribute to goal conflict among institutions if they struggle to balance competing priorities.

Our research also contributes to a growing body of literature on organizational learning and institutional change, demonstrating the challenge faced by colleges in reviewing and responding to student outcome data and finding resources to implement solutions. Using Huber’s (1991) framework on the five methods for acquiring knowledge during the organizational learning process, we found that the most common approach in our survey was “searching and saving” where members search for solutions to problems from their environment. Institutions most commonly identified solutions from external sources such as conferences held by professional associations. Additionally, many college leaders also reported relying on internal knowledge based on past experiences at their institution, which provides support for the experiential learning method. The method used least frequently was “grafting” by hiring new members with knowledge, which is likely attributable to the high cost of additional personnel. Although hiring new staff members with knowledge of potential solutions is the least used method, it could be one of the tools necessary for knowledge acquisition as defined by Huber (1991). As referenced in the previous literature, the challenges with reviewing and responding to data align with the issue of being able to build capacity to make effective changes. Without this ability to build capacity, institutions will likely continue to face challenges related to resources and knowledge acquisition when attempting to make meaningful changes.

Further examining our results from the perspective of organizational learning, the responses from the survey indicate that it would be unlikely and difficult for double-loop learning to occur in large-scale changes such as this statewide reform. With the strain on resources, both time and financial, some institutions struggle to make meaningful change where this type of learning occurs. Additionally, respondents noted that second to finding resources, the most challenging issue when reviewing and responding to data was understanding the underlying issues, a necessary component in the double-loop learning process.

The organizational change process was also hindered due to the lack of resources, as the reform was largely an unfunded mandate that did not provide additional financial support to make rapid changes. While financial incentives were included in the state’s performance-based funding system, this did not occur until two years after the reform was implemented and support was also needed upfront to make the changes in order to achieve the level of performance needed to receive the incentives. While institutions used a variety of methods to identify potential solutions to improving student outcomes, leaders noted that one of the greatest challenges was finding the resources to implement these solutions.

The results of this study have several implications that policymakers and state agency staff need to consider when designing large-scale or statewide educational reforms. First, policymakers should ensure that accountability metrics are aligned with the reform’s intent and the data can be used to support the organizational learning process. Technical assistance with collecting, reviewing, and responding to data, particularly data used in accountability reports, is an area where many respondents found support lacking. Second, state agency staff should ensure that there are adequate opportunities for staff involved with the reform’s implementation to attend professional conferences, and make readily available research about practices to improve student success. Institutional leaders noted that these “searching and saving” techniques were used most extensively when identifying solutions for improving student outcomes. Third, policymakers need to provide the time and financial resources needed by college leaders to implement the solutions that may lead to institutional change. While Florida provided financial incentives through the Performance-Based Funding Incentive, other resources were necessary during the early stages of implementation to allow institutions to respond to data and make changes geared toward improvement. Giving consideration to these types of factors will help to encourage organizational learning and lead to meaningful change in large-scale educational reforms.
